# Effect of Adding Carbon Nanotubes on Corrosion Rates and Steel-Concrete Bond

**DOI:** 10.1038/s41598-019-42761-2

**Published:** 2019-04-18

**Authors:** Ahmed Hassan, Hala Elkady, Ibrahim G. Shaaban

**Affiliations:** 1 Civil Engineering Department, Beni-Suef University, Beni Suef, Egypt; 2 Civil Engineering Department, National Research Centre, Cairo, Egypt; 3 University of Liverpool, Liverpool, UK; 4 Civil Engineering and Built Environment, University of West London, London, UK

**Keywords:** Civil engineering, Nanoscale materials

## Abstract

This paper presents a continuation of the evaluation of utilizing Nano Carbon Tubes (CNTs) in reinforced concrete (CNT-CRETE). The compressive, tensile and bond strengths of the samples with and without CNTs were investigated. Scanning Electron Microscope (SME) was utilized to study the microstructure of the prepared samples. In addition, the corrosion resistance of CNT-CRETE, was measured and compared to traditional concrete. Four mixes were prepared, with 0.01%, 0.02%, and 0.03%, CNTs by weight of cement, along with a control mix without CNTs. The results of the experimental work showed that adding CNTs led to an increase in the compressive, tensile and bond strengths of specimens compared to those of the control specimen. SEM examination for control and CNTs specimens showed that CNTs specimen were well structured compared with the control specimen and this affirms that CNTs act as bridges across micro cracks, which explains the improvement in mechanical properties. The diameter of steel bars played a significant role in failure mechanism for pull-out testing and corrosion resistance. In general, adding CNTs to the concrete mix increased the rate of corrosion for steel bars within the low risk limits. Benefits from using CNTs were limited to moderate. Mineral/chemical admixtures or fibers provide better improvements in the mechanical properties of concrete without the problems associated with dispersing CNTs and the health hazard of handling a Nano material.

## Introduction

Bond strength of steel reinforcement in concrete structures is one of the important factors in structural design^1,2^. Several models were developed for bond strength, taking into consideration the effect of the bar diameter, embedded bar length in concrete, concrete strength, cover thickness and crack spacing are cited in literature^3–8^. ACI 408 committee^9^ explained that the bond between steel and concrete occurs by chemical adhesion between both materials. Frictional forces result from the interface between concrete & steel, and mechanical bearing of the steel ribs against the concrete surface are responsible of carrying the tensile forces in concrete section. The development length of steel bars, required to ensure adequate transfer of stresses, can be calculated using equations specified in various Building Codes^10,11^. Corrosion of reinforcement, as a result of exposure of reinforced concrete to aggressive environments, has a negative effect on the bond strength of steel reinforcement in structural concrete. With further exposure, the increased volume of corrosion products may cause the concrete cover to crack, and will definitely result in bond strength degradation^12–14^.

The bond strength loss for corroded reinforcing bar is much more critical than the cross-sectional area loss^15^. The bond behaviour of steel reinforcement in reinforced concrete elements, including the ultimate bond strength, the load-slip relationships and the effect of different corrosion rates were studied in literature^16–18^. The bond behaviour between normal aggregate concrete or recycled aggregate concrete and corroded steel bars was also investigated^19^. The partly corroded steel bars exhibit different bond behaviour from that of the whole-surface corroded bars^20^. The effect of corrosion on stiffness and maximum strength of bond was expressed as a function of corrosion percentage^21^. A frictional model was developed and a damage model for bond strength assuming a scalar damage parameter to account for the bond strength loss in the full development of slip^22^ while a simple probabilistic bond strength model considering corrosion using a multivariable regression analysis and based on a comprehensive database was proposed^23^. An empirical model were developed for the ultimate bond strength by evaluating bond strength in different concrete mixes, different covers and different corrosion levels^24^.

Concrete as a cementitious composite material has a complex network of binding particles known as calcium silicate hydrate (C-S-H). Using Carbon Nanotubes (CNTs) improves the mechanical properties of concrete, as reported in the literature^25^. CNTs shall interact most intimately with CSH due to their Nano-scale characteristics. The interface or CNTs with C-S-H is achieved by the large number of atoms present at the nanotube surface. CNTs have high flexibility, and its microstructure shape as tubes is capable of bending in circles and forming bridges crossing the micro and Nano-cracks developed in the cement composites, which in turn, increases the strength of the cement composites^26^. CNTs can be widely distributed in the concrete paste, and as a Nano material, its interaction with the paste is more intense than that of the larger fibers. CNTs possess a high modulus of elasticity, tensile strength, and yield strain. Such properties make CNTs an attractive reinforcing material for concrete. The unique features offered by CNTs provide an excellent opportunity to develop CNTs-reinforced structural concrete^27^. Addition of CNTs to the concrete mix and repair mixtures was studied in literature^28,29^. CNTs improve cement composites in tension due to its ability to prevent the initiation of micro cracks in the cement matrix^30^.

## Research Significance

Members of this research group have been investigating the capabilities of CNT-CRETE^29^. Earlier published work was devoted to the effect of adding Nano silica materials in reinforced concrete mixes on different mechanical properties including bond and corrosion resistance^31^. In a recent publication^32^ the effect of CNTs on the bond strength of concrete was also studied. However, their mixes had compressive strengths in excess of 46.2 MPa, classifying these as high strength concrete. In addition, they added high percentages of CNTs, namely 0.05 and 0.1%. They only used one bar diameter (12 mm), to prepare the samples. In the current study, the maximum strength of the mixes was 35 MPa in the range of typical normal strength concrete and the content of CNTs was limited to a maximum of 0.03% by weight of cement. Therefore, the current investigation will add to the body of knowledge concerning the effect of a moderate percentage of CNTs on the bond strength of normal strength concrete, utilizing two steel bar diameters (12 and 16 mm) for the bond strength test. The authors did not cite any other publications presenting results of experimental work assessing the bond strength of CNT-CRETE.

The results in the literature on the effect CNTs on the corrosion rate of steel embedded in mortar samples are contradictory. The addition of CNT caused higher corrosion intensities for cement mortars with up to 0.5% CNT subject to simulated sea water and accelerated carbonation^33^. On the other hand, Konsta-Gdoutos *et al*.^34^ reported that the inclusion of 0.1 wt% CNTs decrease the corrosion rate and significantly increased the resistance to corrosion by delaying the onset of the corrosion reaction. All previous studies on the effect of CNTs on corrosion rates were conducted on mortar samples. The authors cited no corrosion studies conducted on CNT-CRETE. In addition, the current study compared the benefits from using CNTs in concrete with those attained by using chemical/mineral admixtures or fibers to provide practical guidance to civil engineers.

## Experimental Program

### Material Properties, Mix Proportions and Mix Procedure

**Figure 1:**
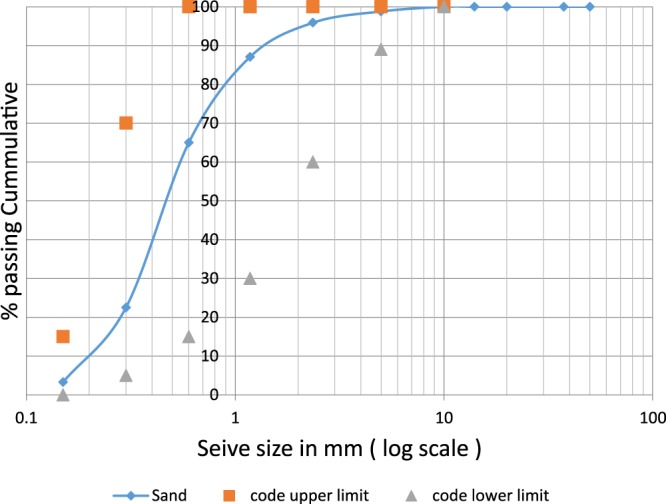
Fine Aggregates Sieve Analysis According to Limits^53^.

**Figure 2:**
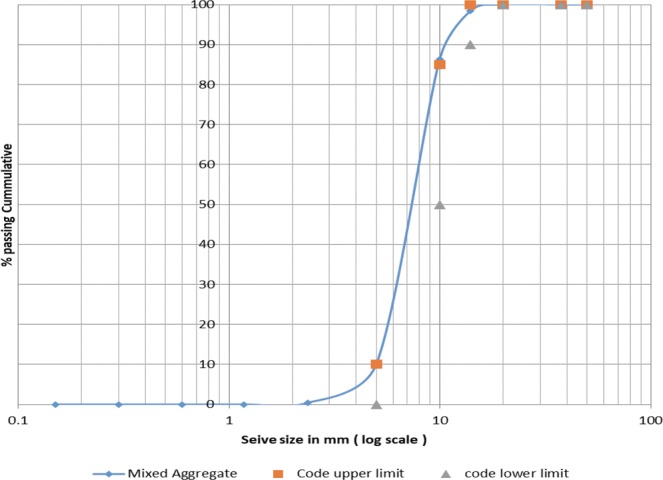
Dolomite Aggregates Sieve Analysis According to Limits^53^.

**Figure 3:**
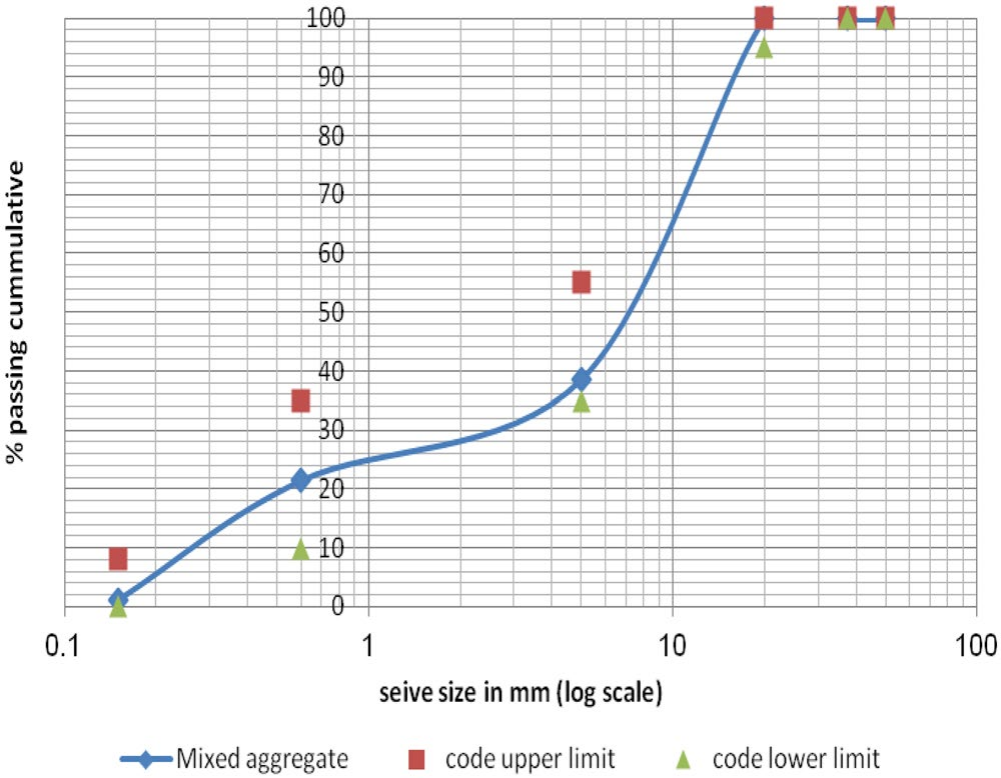
Mixed Aggregates Sieve Analysis According to Limits^53^.

Ordinary Portland Cement (OPC) according to ASTM C150 standard was used in all mixes of this study. Cement physical and chemical properties are shown in Table 1. Sieve analysis for fine aggregates used in this study is shown in Fig. 1. Crushed dolomite of maximum size of 12 mm and specific gravity of 2.96 g/cm^3^ was used as coarse aggregate; its sieve analysis is given in Fig. 2. Figure 3 shows the sieve analysis of the mixed aggregates. Potable water is used in mixing. Mix proportions of the control mix are given in Table 2. The type of superplasticizer was chosen due to its electrostatic-steric behavior which has a significant effect on CNTs dispersion. Local multi-walled carbon nanotubes (MWCNTs) with diameters of 8–22 nm were used throughout the work^35^.

**Table 1:** Ordinary Portland Cement Properties.

Element	SiO_2_	Al_2_O_3_	Fe_2_O_3_	CaO	MgO	SO_3_	Na_2_O	K_2_O	L.O.I
Cement	20.13	5.32	3.61	61.63	2.39	2.87	0.37	0.13	1.96
Property					Result				
Compressive strength (3-days)					158 kg/cm^2^				
Compressive strength (7-days)					kg/cm^2^

**Table 2:** Concrete Mix Proportions for the Control Mix.

Fc (MPa)	Water	Cement (kg/m^3^)	Coarse Aggregate (kg/m^3^)	Fine Aggregate (kg/m^3^)	Superplasticiser(G) (kg/m^3^)
30	164	335	1195	644	0.9

**Figure 4:**
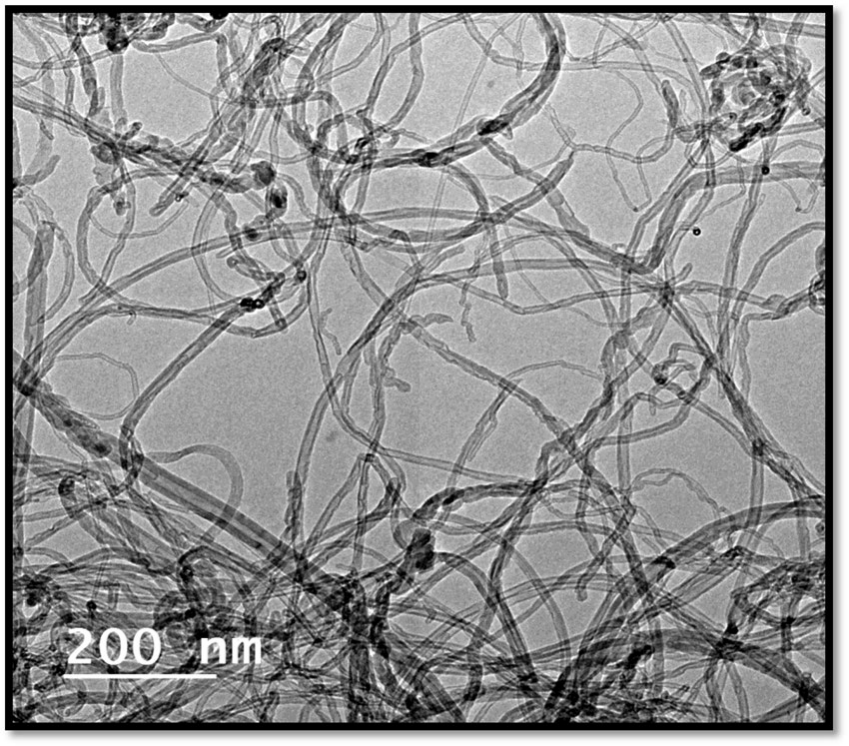
CNTs TEM MICROGRAPH.

**Figure 5:**
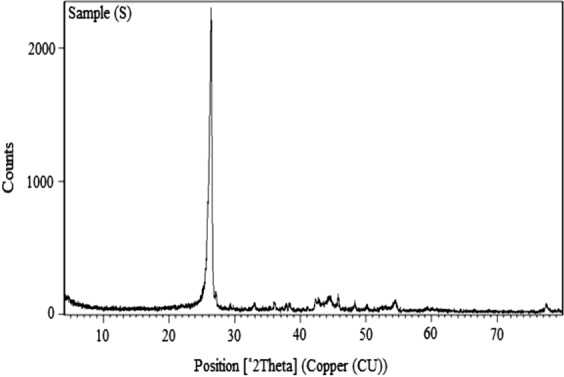
XRD OF CNTs.

The representative transmission electron microscope micrograph and XRD of the CNTs are shown in Figs 4 and 5, respectively. High tensile steel of 360 MPa yield stress were used for the bond strength test. The designed concrete strength was 30 MPa. Three different percentages of CNTs, 0.01%, 0.02%, and 0.03% replacement by cement weight were add to concrete mixes. The dry constituent materials were mixed using a concrete rotating drum mixer with a capacity of 0.125 m^3^ for 2 minutes, then the mixture of the tap water, CNTs was added to the concrete mixer and mixing was continued for another two minutes.

### Test Specimens Preparation

**Figure 6:**
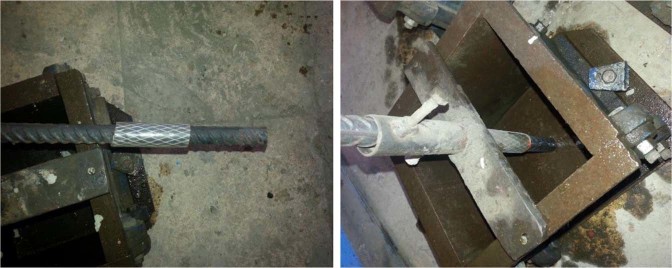
Moulds and testing cubes with steel rods inserted.

Twelve concrete cubes of dimensions 150 mm × 150 mm × 150 mm and twelve cylinders 150$\varnothing$ mm × 300 mm were cast to determine the compressive strength according to BS EN 12390–3^36^ and tensile strength of concrete after 28 days according to ASTM C 496^37^, respectively. Three specimens for control concrete mix and three specimens for each concrete mix contained CNTs at percentages of 0.01%, 0.02%, and 0.03% by weight of cement. The well-mixed concrete mixture, for each batch was poured into moulds to form three cubes of size 150 mm × 150 mm × 150 mm for the compressive strength testing and three cylinders 150$\varnothing$ mm × 300 mm to calculate tensile strength for every mix. After being demoulded at the age of one day, all specimens were cured in water at 25 °C till the age of testing. Concrete cubes of dimensions 150 mm × 150 mm × 150 mm were cast and high-grade steel rods were installed in the moulds prior to casting as shown in Fig. 6. Specimens were demoulded after 24 hours and cured at room temperature in normal pure water until the day of testing. The studied specimens were divided into four groups, each group consisted of four different mixes and three cube specimens were cast of each mix as reported in Table 3. Therefore, each group contains twelve cubes and the total was forty eight cubes. The steel rods installed in Groups 1 and 3 were 12 mm diameter while those installed in moulds of Groups 2 and 4 were 16 mm in diameter. The CNTs addition of the mixes was 0.01%, 0.02% and 0.03% for each Group. The bars were embedded into the full length of the concrete cube specimen.

**Table 3:** Details of test specimens for bond pull out and corrosion tests.

Group no.	Specimen Designation	CNTs	Steel diameter	Test type	Time
G1	12 mm	0	12 mm	Pull out	
	0.01% 12 mm	0.01%			
	0.02% 12 mm	0.02%			
	0.03% 12 mm	0.03%			
G2	16 mm	0	16 mm		
	0.01% 16 mm	0.01%			
	0.02% 16 mm	0.02%			
	0.03% 16 mm	0.03%			
G3	12 mm	0	12 mm	Accelerated Corrosion Test	15–30 DAYS
	0.01% 12 mm	0.01%			
	0.02% 12 mm	0.02%			
	0.03% 12 mm	0.03%			
G4	16 mm	0	16 mm		
	0.01% 16 mm	0.01%			
	0.02% 16 mm	0.02%			
	0.03% 16 mm	0.03%			

Half of the length of the embedded part of the bar will be de-bonded using polyvinyl chloride tubing to ensure the bond slip failure dominates over other types of failure. The cube specimens are de-moulded after 24 hours and cured at room temperature in normal pure water until the day of testing.

### Testing Setup

#### Compressive, Tensile and SME Tests

A 1000 KN universal testing machine was used for the compressive, tensile and bond strength testing of the RC and CNT-CRETE specimens. Scanning electron microscopy (SEM) made in a JEOL JSM6300 from Oxford Instruments. The secondary electron images were obtained in samples coated with Au and using a voltage of 20 kV.

#### Measurement of Corrosion Rates

**Figure 7:**
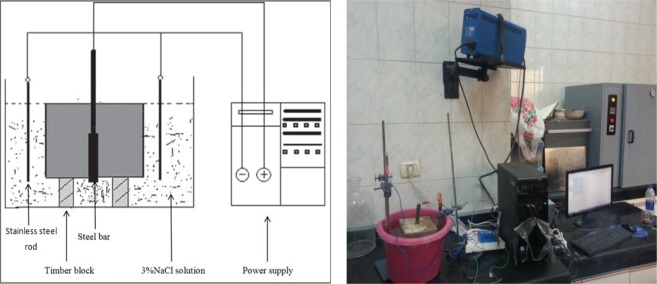
Corrosion or Electrochemical Test Set Up.

The specimens were immersed in a 3% NaCl solution by weight of water. The impressed current direction is to be adjusted such that the reinforcing steel served as the anode while a stainless steel rod positioned in the tank was acting as a cathode. In order to establish different levels of reinforcement corrosion, accelerated corrosion time was set over 1, 7 and 15 days. A schematic representation of the electrochemical test setup is shown in Fig. 7 along with the laboratory photo. Table 4 outlines the full details of corrosion apparatus used in this study.

**Table 4:** Corrosion apparatus (SP-150) properties and specifications.

Weight	7.2 Kg
External dimension	335 × 435 × 95
Input voltage range	90 to 264 Vac
Power	65 W max
Frequency	50 to 60 Hz
Fuses (Neutral + Phase)	2 × 2 AF, cold start-250 VAC (5 × 20 mm)
Output	±10 Vdc/800 mA or ±10 Vdc/240 mA with Low-Current option
Operating Temperature	10 °C to +40 °C
Storage Temperature	0 °C to +50 °C
Pollution degree	1 (no pollution)
Humidity	10% to 80% non-condensing
Cooling	Internal DC Fans
Vibration	not specified
Choke	not specified

#### Pull out test

**Figure 8:**
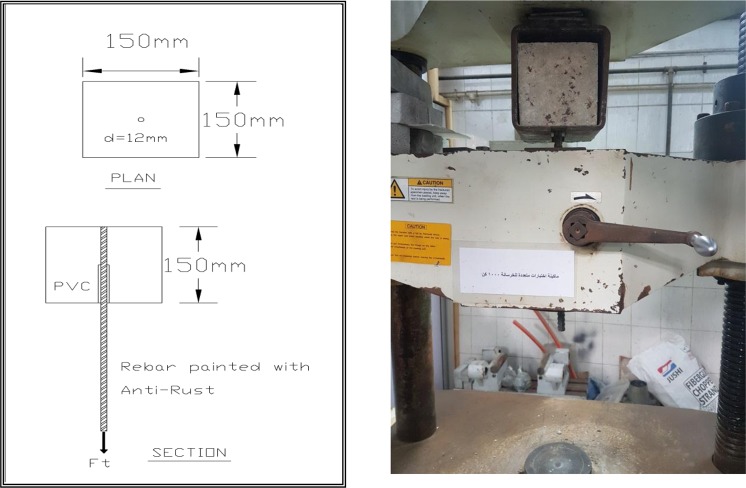
Pull Out Bond/Strength Test Setup.

Figure 6 shows details of the pullout test mould and specimens.Test setup for pull out test is shown in Fig. 8. Vertical displacements of the tested cubes were recorded automatically. The pull put test was carried out at after 28 day of casting.

## Results and Discussion

### Compressive and Tensile Strength Tests

The compressive strength results at 7 and 28 days and the splitting tensile strength are reported in Table 5. It can be seen that the addition of CNTs to the concrete mix led to an increase in the compressive strength of specimens compared to that of the control specimen. The range was approximately from 7% to 20%, for 0.01% to 0.03% addition of CNTs. An increase of up to 20% was also reported^33,34^ when up to 0.06% CNTs were included in concrete mixes. Hawreen and Bogas^32^, argued that the increase in compressive strength of CNT-CRETE compared to normal concretedue to the CNTs filler, nucleation and bridging effects. They also found that the increase ranges between 3 and 21% for their mixes containing different types of CNTs at 0.05 and 0.1% by weight of cement. They considered this to be a modest improvement that can be achieved by other means. Moreover, they attributed this modest improvement to the CNTs’ inability to significantly enhance the quality of the aggregate–paste bond. In another publication, when 0.13% CNTs were used in mortar, the improvement in compressive strength was 37% and 20% after 1 day and 28 days of curing, respectively^38^. So it appears that CNTs is more beneficial at the early ages when the mortar is weak.

**Table 5:** Effect of CNTs on the compressive and splitting tensile strengths of concrete.

Concrete Type	Compressive Strength (MPa)		Tensile Strength (MPa)
	7 days	28 days	28 days
Control	24	29	3.0
0.01% CNTs	26	31.2	3.3
0.02% CNTs	28	33	3.5
0.03% CNTs	29.7	35	3.6

For comparison, Amudhavalli and Mathew^39^ found that adding 15% silica fume to a 38.3 MPa mix, lead to 23.5% increase in compressive strength. In another research^40^, it was found that 8% silica fume increased the compressive strength by 18% when added to a 44 MPa control concrete. Just adding 0.5% superplasticiser^41^, whilst keeping all other proportions constant increased the compressive strength of a 34 MPa mix by 40%.

Adding of CNTs into concrete mix led to an increase in the splitting tensile strength of studied specimens compared to that of the control specimen. The tensile strength in the specimens including CNTs were higher than that of the control mix by a range from 10 and 20%, at CNTs dosages of 0.01% to 0.03%, respectively.

Qissab and Abbas^42^ found that the splitting tensile strength was increased by 3.1 to 37.5% when up 0.06% of CNTs were added to the samples. Their samples contained both long and short CNTs. They reported that SME images show breakage of CNTs along the fractured surfaces of the samples. No CNTs were pulled from the cement paste during the fracture. Hunashyal *et al*.^43^ found that the mortar samples with 0.5% CNTs exhibited an increase of 19% in direct tensile strength compared to plain mortar samples. They reported less strain at failure of CNTs samples. For comparison, the inclusion of 1% steel fibers (having a tensile strength of 2000 MPa) into a w/c = 0.55 concrete mix, lead to an increase in the splitting tensile strength by 36.3%^44^. The increase was higher for mixes with lower w/c, and the samples exhibited higher ductility due to fiber inclusion. In another investigation^45^, it was observed that addition of 0.5%, 50 mm copper coated crimped round steel fiber having aspect ratio 53.85 to concrete having a compressive strength of 25 MPa resulted in an increase in the splitting tensile strength by 61.1%. Shah *et al*.^41^ found that adding 1.5% superplasticiser to a 34 MPa mix, whilst keeping all other ingredients the same, increased its splitting tensile strength by more than 120%.

### Effect of CNTs on Microstructure of Concrete

**Figure 9:**
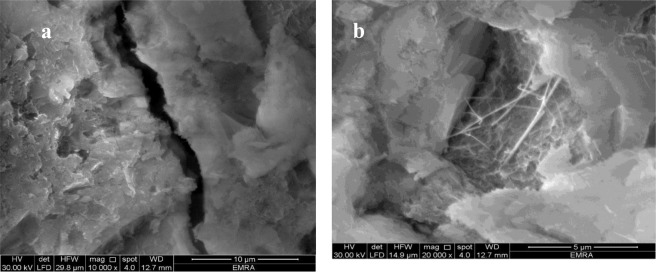
SEM images for (**a**) Control cubes and (**b**) CNTs cubes.

Figure 9a,b show the results obtained from scanning electron microscopy analysis for control and CNTs specimens, respectively. It can be seen from the figures that CNTs specimen were well structured (see Fig. 9b) compared with the control specimen, which exhibited at least one major crack in its structure (see Fig. 9a). Some studies reported that, the new composite material (partial cement replacement with CNTs) is well-structured^46^. However, there is evidence^47^ from extensive SEM observations that despite the best efforts to maintain the dispersion of nano filaments within the aqueous solution, combining a well-dispersed aqueous mixture with cement and water resulted in a relatively poor dispersion within the hydrated cement paste.

### Effect of CNTs on Corrosion Rate

**Figure 10:**
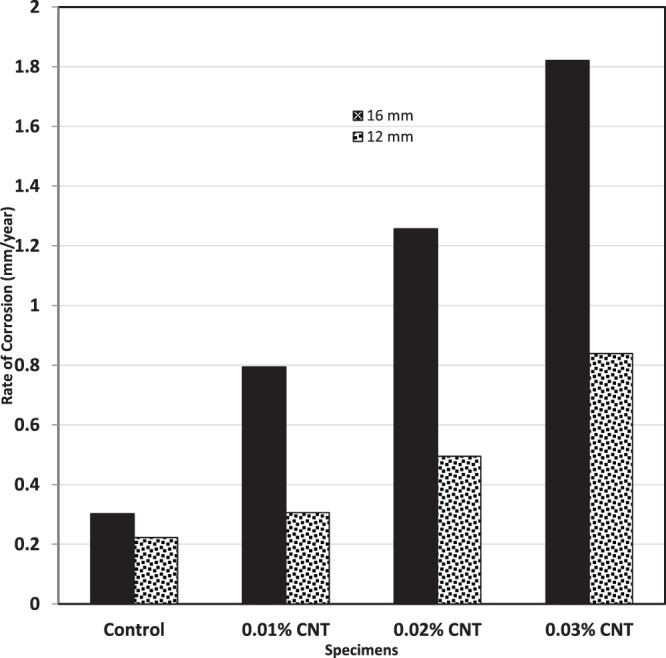
Effect of adding CNTs to Corrosion Rates of Steel in Concrete.

The test results in this study show that corrosion rates are affected by diameters of steel bars. For example, steel reinforcement of 16 mm diameter had a lower corrosion resistance than that of steel reinforcement of diameter 12 mm. It was observed that addition of CNTs increased the rate of corrosion for steel reinforcement especially for 16 mm diameter steel reinforcement as shown in Fig. 10. The maximum rate of corrosion was 1.85. This value was considered as low risk according to codes specifications^10,11^. The increase of corrosion rate by increasing CNTs percent agrees with the findings of Konsta-Gdoutos *et al*.^34^ who reported that adding 0.5 wt% CNTs mortar mix exhibited higher corrosion rate values than those for 0.1 wt% CNTs samples. However, Konsta-Gdoutos *et al*.^34^ could not explain their results because when they added Nano filler material (CNFs) to the 0.1 wt% CNTs, the onset of the corrosion was delayed. In the authors view, the delay in the onset of corrosion is due to a marginal decrease in permeability leading to reduction in the ingress of aggressive agents. However, the increased tendency to corrosion needs further investigation.

Since, corrosion is an electrochemical process; the effect of CNTs on corrosion can be explained from both an electrical and chemical perspectives. It was found^48^ that adding multi walled CNTs, like those used in the current investigation, resulted in a significant reduction in the resistivity of cement paste. The reduction in resistivity was more for pastes with higher percentages of CNTs. This reduction in resistivity means an increase in electric current conductivity leading to higher rates of corrosion. In another publication^49^, it was observed that: “the addition of CNT to the cement matrix could imply the development of higher levels of corrosion in aggressive conditions, such as carbonation and contamination by chloride ions”. This was explained from a chemical perspective by stating that: “The union of two different conductive materials with different nobility implies that the less noble tends to develop higher corrosion rates than the same element without such electrical contact. On the other hand, the material with higher nobility develops lower corrosion rates. The former argument is consistent because the electrons of the less noble material (steel) will cause cathodic protection on the other one (CNT). For this reason, a higher content of CNTs implies higher levels of the Icorr values.” In simple words increased corrosion rates of steel in cement composites with CNTs, since steel is less noble than CNTs. This explains the results of the current and previous investigations on the corrosion in the presence of CNTs.

### Effect of Steel Diameter on Pull out Test

**Figure 11:**
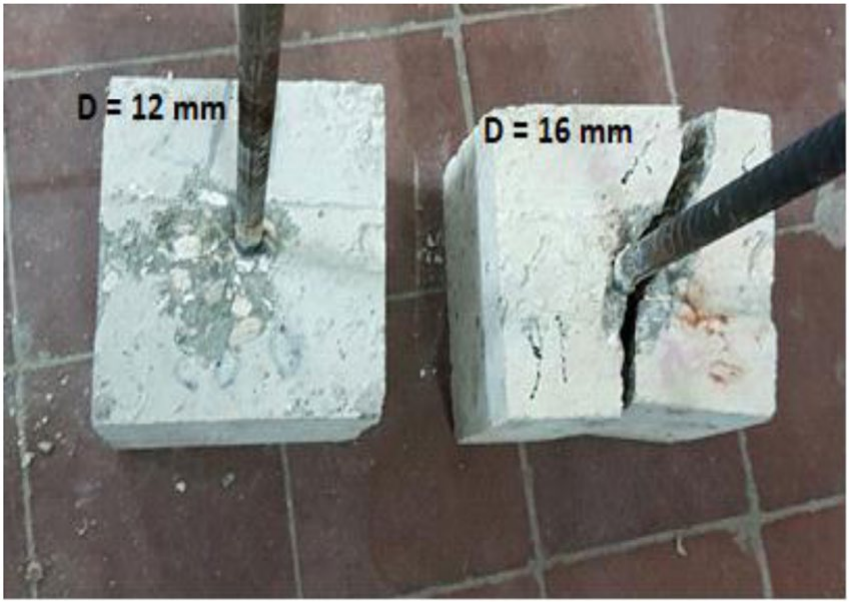
Effect of steel diameter on pull out test mode of failure.

**Figure 12:**
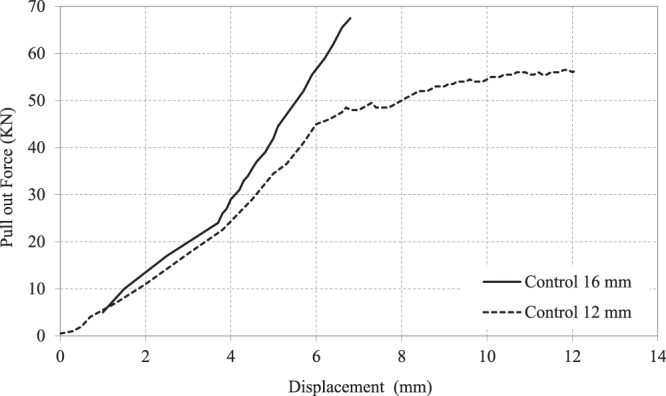
Effect of steel diameter on Pull out force–Displacement curve in control samples without CNTs.

Pull-out or splitting is the common failure mode of steel-concrete bond test. The diameter of steel bars plays a significant role in failure mechanism. Failure of steel bars of 12 mm diameter was by pull out mode whereby the bar pulled out slowly as the load dropped steadily as shown in Fig. 11. Nevertheless, minimal splitting was noticed along the top surface of the test bar 12 mm. On the other hand, splitting failure occurred when tests were carried out for specimens of steel reinforcement with bars of diameter 16 mm. Pull out force versus displacement responses (slip of the bar at free end) relationships are shown in Fig. 12 for control specimens. Following the formation of internal cracks and initiation of de-bonding, the stiffness of the ascending curves gradually softens until reaching a maximum pull-out force value. The ultimate force and corresponding slip were, however, directly dependent on the diameter of steel bar. The total force of pull out forces for steel bars of diameters 12 mm and 16 mm were 56 KN and 67 KN, respectively. For a similar grade concrete, it has been reported^50^ that the bond pull out force was 17 and 20 KN for 16 mm and 20 mm bars, respectively. However, the embedment length for those samples was only 65 mm, whereas in the current study it was 150 mm, which explains the higher forces reported in the current study. Bompa, and Elghazouli^51^ measured pullout forces of 118 and 189 KN for steel bars of 16 and 20 mm diameter. However, their samples had a compressive strength of 78.6 MPa, which explains why they recorded higher pullout forces.

### Effect CNTs on Steel-Concrete Bond for Different Steel Diameters for Pull out Test

**Figure 13:**
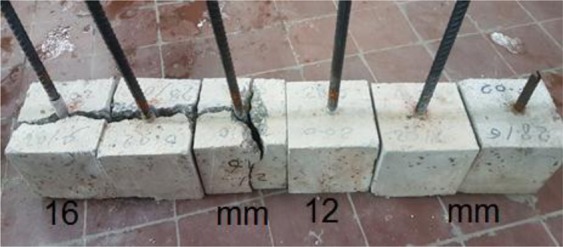
Effect of Steel Diameter onFailure Mechanism in CNT-CRETE.

**Figure 14:**
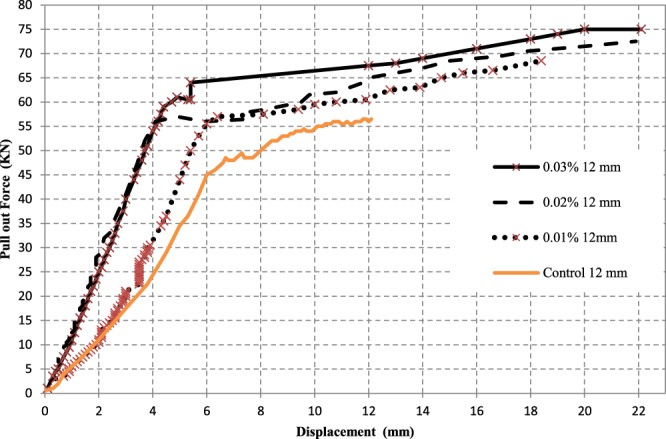
Effect of CNTs % on Pull out Force – Displacement curves for the pull out test of 12 mm steel bars.

Figure 13 shows the deformed bar specimens in concrete cubes and their failure mode as a pull-out failure and splitting failure. The control mix showed two types of failure modes: pull-out failure and splitting failure for steel reinforcement bars of diameters 12 mm and 16 mm, respectively. Three radial cracks appeared on the front loaded-end face for bars of diameter 16 mm after adding more than 0.02% CNTs, which extended on the two sides and top faces, as shown in Fig. 13. Figure 14 shows pull out force versus displacement responses (slip of the bar at free end) relationships for specimens of bars diameters of 12 mm and including CNTs.

**Figure 15:**
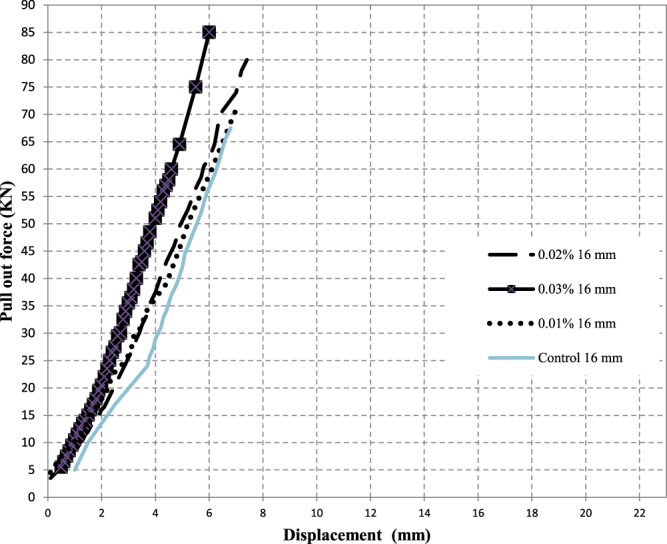
Effect of CNTs % on Pull out Force – Displacement curves for the pull out test of 16 mm steel bars.

It can be seen that all curves yielded initially a stiff response that varied up to around 13% of the maximum force and beyond which the bars free end started to slip noticeably. This was explained earlier in the literature that, in this region, the bond resistance consists mainly of chemical adhesion and friction at the interface between the bar and concrete matrix^10^. Figure 15 shows the pull out force – displacement relationships for steel reinforcement of bar diameter 16 mm. It was found that the bond force of control specimen is less than that of the CNTs concrete mix. In addition, adding CNTs improves the bond strength of steel reinforcement bars of 12 mm and 16 mm diameters by 36% and 21%, respectively.

In another investigation^50^, 0.5% hooked end steel fibers increased the pull out force in a 44 MPa mix by 17 and 42% for 16 and 20 mm bars. Other reports^52^, found that with higher hooked end fiber contents, up to 3% by volume, in a w/c = 0.32 concrete (designed to have a compressive strength of 40 MPa), the pull out force of a 16 mm bar was increased by 48%.

## Conclusions

The experimental work carried out in this investigation assessed the effect of adding CNTs on the corrosion rates and pullout forces in concrete cubes. The main conclusions can be drawn as follows:

1. It was found that adding CNTs to concrete led to some increase in both of the compressive and tensile strength of specimens compared to that of the regular concrete control specimens. Adding 0.01% to 0.03% CNTs led to an increase ranges approximately from 7 to 20% for compressive strength, and from 10 to 20%, for tensile strength.
2. Results obtained from scanning electron microscopy analysis for control and CNTs specimens show that CNTs specimen is well structured and dispersed compared with the control specimen, and this affirms that CNTs act as bridges across micro cracks, which is helpful to improve the bond ability.
3. It was found that the ultimate force and corresponding slip were directly dependent on the diameter of steel bar. The total force of pull out forces for steel bars of diameters 12 mm and 16 mm were 56 KN and 67 KN, respectively. This confirmed the dominating role of the lateral surface area of the steel bars in failure mechanism for pull-out testing.
4. CNTs concrete has a better bond strength performance compared to a corresponding concrete specimen. In addition, adding CNTs improves the bond strength of steel reinforcement bars of 12 mm and 16 mm diameters by 36% and 21%, respectively.
5. Steel reinforcement of 16 mm diameter has a lower corrosion resistance than that of steel reinforcement of diameter 12 mm. The maximum rate of corrosion in the assessed samples did not exceed 1.85% and this value is still considered as low risk according to ACI 318. In general, the CNT-CRETE has higher corrosion tendency compared to plain concrete. The increase of corrosion rate in the presence of CNTs was explained from both the electrical and chemical perspectives.
6. The benefits of CNTs inclusion on compressive, tensile and bond strengths can be achieved, or in many cases exceeded, by adding chemical admixtures, such as superplasticisers, cement replacement materials, such as silica fume, or steel fibers.
7. Given the effort needed for dispersion prior to mixing, the results of the current and previous investigations on the topic, and the health hazards associated with handling nano materials; and the increased tendency to corrosion of CNT-CRETE; the authors are not in favor of using CNTs in concrete to induce mechanical properties improvements.
